# Predicting novel genomic regions linked to genetic disorders using GWAS and chromosome conformation data – a case study of schizophrenia

**DOI:** 10.1038/s41598-019-54514-2

**Published:** 2019-11-29

**Authors:** Daniel S. Buxton, Declan J. Batten, Jonathan J. Crofts, Nadia Chuzhanova

**Affiliations:** School of Science and Technology, Nottingham Trent University, Clifton Lane, Nottingham NG11 8NS UK

**Keywords:** Predictive markers, Computational models

## Abstract

Genome-wide association studies identified numerous loci harbouring single nucleotide polymorphisms (SNPs) associated with various human diseases, although the causal role of many of them remains unknown. In this paper, we postulate that co-location and shared biological function of novel genes with genes known to associate with a specific phenotype make them potential candidates linked to the same phenotype (“guilt-by-proxy”). We propose a novel network-based approach for predicting candidate genes/genomic regions utilising the knowledge of the 3D architecture of the human genome and GWAS data. As a case study we used a well-studied polygenic disorder ‒ schizophrenia ‒ for which we compiled a comprehensive dataset of SNPs. Our approach revealed 634 novel regions covering ~398 Mb of the human genome and harbouring ~9000 genes. Using various network measures and enrichment analysis, we identified subsets of genes and investigated the plausibility of these genes/regions having an association with schizophrenia using literature search and bioinformatics resources. We identified several genes/regions with previously reported associations with schizophrenia, thus providing proof-of-concept, as well as novel candidates with no prior known associations. This approach has the potential to identify novel genes/genomic regions linked to other polygenic disorders and provide means of aggregating genes/SNPs for further investigation.

## Introduction

Decades long genome-wide association studies (GWASs) have identified numerous loci harbouring single nucleotide polymorphisms (SNPs) and other genetic variants associated with a range of human diseases, although the causal role of many of them remains unknown. *In silico* ascertainment of SNPs and SNP-harbouring loci is hampered by several factors including their location and small effect size of SNPs. It is known that approximately 93% of disease-associated variants reside outside protein coding regions^1^, potentially within unknown regulatory elements. These regulatory elements do not necessarily target the nearest gene(s) in the vicinity but may reside at considerable distances from the genes they regulate [reviewed in ref. ^2^]. Indeed, it was recently shown that only 14% of SNPs in non-coding regions target nearest genes^3^, prompting a need for more accurate ways of identifying SNP-target gene pairs, instead of a simple assignment of a SNP to the nearest gene. In addition, many diseases are polygenic, relying on the cooperation of small effect size SNPs in more than one gene, in order for the disease phenotype to exist. Identification of these relevant sets of genes/SNPs and, most importantly, providing a plausible biological explanation for their cooperation is not a trivial undertaking. SNPs are usually aggregated either at the level of genes or a set of genes which share a known biological function(s) or pathway. To assess the joint effect of groups of SNPs, various set-based approaches, not requiring individual genotype data, have been developed (e.g. ref. ^4^). Another group of methods is based on polygenic risk scores^5^ that are usually used to predict phenotype likelihood by assessing the joint effect of a group of SNPs. The latter approaches usually require two samples – a discovery sample, comprising GWAS summary statistics, and an independent target sample with known individual genotype data, which may not be readily available.

In this paper we postulate that co-location of novel genes with genes, known to be associated with a specific phenotype, and their enrichment in the same biological function or pathway as known genes, make them good candidates for novel genes, linked to the same phenotype (“guilt-by-proxy”). We hypothesise that SNPs residing within these groups of co-located genes, comprising both novel and known “guilty” genes, may contribute to the observed phenotype either individually (when a single common SNP residing in one of these genes could cause a phenotype), or collectively (when SNPs residing in several functionally-related genes may have an additive effect on the observed phenotype), or selectively (when SNPs exhibit genome-wide significance only in a smaller and possibly more homogeneous subgroup of patients stratified by their origin, age, gender, etc.). We further postulate that both non-coding SNPs and their potential target genes also reside within co-located loci.

To find novel genomic regions co-located with loci that have proven association with a given phenotype, we propose a conceptually new approach to identification of these co-located regions that utilises the knowledge of the 3D architecture of the human genome and is based on *in silico* analysis of networks of co-located loci. As a case study for the proposed approach, we investigate the well-studied disorder – schizophrenia (SCZ). We further expanded a dataset of SNPs generated by the Psychiatric Genomics Consortium (PGC; refs. ^6,7^) by SNPs found via review of literature and the use of various bioinformatics resources. As a result, 346 genes linked to schizophrenia and 1,252,901 SNPs were identified although not a single SNP was found to have a large enough effect size to claim sole responsibility for SCZ development. Hence, SCZ is considered to be a polygenic disorder that relies on the cooperation of small effect size SNPs in order for the disease phenotype to exist^8^.

To assess the co-location (or proximity) of two distinct loci, not necessarily on the same chromosome, we used datasets of interaction frequencies generated by various chromosome conformation capture techniques^9–11^ and available for a range of cell lines. Note that the frequency of intra- and inter-chromosomal interactions between two loci is inversely proportional to their closeness within the cell nucleus.

We used brain-specific expression quantitative trait loci (eQTL) data from Braineac/Brain eQTL Almanac database^12^ to choose the most relevant cell line, which emulates 3D interactions in brain tissues and for which chromosome conformation data was available at the time when this study was initiated. A network of interactions was created from the interaction data for this cell line with nodes representing genomic regions, connected by edges, weighted by interaction frequencies. We calculated various network centrality (e.g. degree) measures^13^ and identified communities (i.e. densely connected subnetworks) existing within the network with the aim of revealing influential nodes/regions and close-knit communities within SCZ interaction networks. In the subsequent analysis of candidate regions and subnetworks, we used functional annotation and enrichment analysis tools provided by the Gene Ontology Consortium^14,15^, functional annotation and enrichment analysis tools DAVID^16^, ENCODE data^17^ and compared our results with results obtained by recent SCZ GWAS^18^ and machine-learning approach^19^ to build a compelling case for the phenotypic role of newly found loci that were not present in the expanded PGC dataset but were found solely on the basis of their close proximity to, and shared function with, the “guilty” genes.

## Results

### Selection of the cell line enriched in brain-related eQTL

To determine which cell line most closely resembled interactions in brain tissues, brain eQTL data^12^ was used. The majority (>95%) of *cis*-eQTL pairs recorded in Braineac/Brain eQTL Almanac database^12^ were found residing within fragments that interacted with frequency >0 according to *in situ* Hi-C data^10^ for all cell lines and bin sizes. However, the proportion of *cis*-eQTL pairs residing within strongly interacting fragments (with frequency ≥64, corresponding to ~0.05% of the strongest interactions in the datasets of normalised counts) varied between cell lines. The highest proportion of strong *cis*-eQTL pairs (p ≤ 10^−6^) among these strongly interacting fragments was found for the GM12878 cell line (84%), followed by IMR90 (46%) and HMEC (45.6%). Fisher’s exact test confirmed that there is a significant (p < 10^−398^) enrichment of intra-chromosomal interactions in brain-related eQTL signals for the GM12878 cell line. Moreover, the comparison with the Hi-C data for adult brain cells that recently became available via PsychENCODE resources^19^ showed that ~70% of interactions in brain cells are preserved in the GM12878 cell line. Therefore, GM12878 was clearly the most suitable choice of cell line for emulating 3D interactions in brain tissues. Hence, subsequent approaches utilised only Hi-C data for the GM12878 cell line.

### Properties of original and extended SCZ interaction networks

The SCZ networks of interactions between the extended regions of the 346 SCZ-associated genes (Supplementary Table S1) and/or their regulatory regions amalgamated into 103 non-overlapping extended genomic regions (EGRs), were created as described in Materials and Methods. Only inter-chromosomal interaction with frequency ≥64, corresponding to ~0.05% of the strongest interactions in the datasets of counts normalised using Knight and Ruiz matrix balancing procedure, were considered. As a result, 32 EGRs corresponding to 32 SCZ-associated genes (see Supplementary Table S2) from the set of 346 SCZ-associated genes did not show inter-chromosomal interactions to any other regions; therefore, these isolated nodes were not used in the subsequent analyses. One 100 K region on chromosome 1 (chr1:121,400,001–121,500,000) was found to interact with almost every other 100 K regions. This is a gene-less region which was only partially present in the earlier NCBI36/hg18 assembly. For these reasons the region and all interactions with this region were deleted from the dataset of interactions.

The original network is disconnected and consists of 34 subnetworks (Supplementary Fig. S1). Together with 61 regions harbouring 285 SCZ-associated genes and their regulatory elements, this network contains 85 new regions that exhibit strong interactions with regions harbouring SCZ-associated genes but resided outside of those 108 SCZ risk loci identified by GWAS studies^7^. Comparison of regions corresponding to the nodes in the original network with loci identified by the recent GWAS (ref. ^18^ and Supplementary Table 4 therein) shows that 58 out of the 145 non-overlapping loci identified by meta-analysis of CLOZUK and other available up-to-date PGC GWAS datasets as harbouring significant (p < 5 × 10^−8^) SNPs were also present in the original network. Two loci on chromosome X were not found since all loci residing within this chromosome were excluded from the list of 346 genes. Further, 123 and 89 out of 346 SCZ-associated genes were also found in, respectively, the SCZ high-confidence and risk gene lists (http://resource.psychencode.org) reported in ref. ^19^.

The extended network comprises 700 nodes (regions), covering 419 Mb of the human genome and comprising 71 regions harbouring 314 SCZ-associated genes and 629 novel regions harbouring 8,984 genes. All the 143 loci identified in ref. ^18^ (with the exception of regions on chromosome X as explained above) were present in the extended network. Out of the 302 genes listed in the SCZ high-confidence gene list^19^, 117 genes were found to reside within novel regions, whereas out of the 606 genes listed in the SCZ risk gene list^19^ (without genes overlapping with the SCZ high-confidence gene list), 337 genes were in common.

The extended network consists of one giant component (666 nodes) and 13 disconnected subnetworks (Supplementary Fig. S2), comprising two to six nodes. Two nodes in the giant component, corresponding to regions chr22:38,800,001–43,700,000 (labelled as 22:389–437 in 100 Kb scale) and chrX:20,500,001–21,900,000 (labelled as 23:204–219), have the highest degree, respectively 328 and 304. The region on chromosome 22 together with all connected regions harbours 5513 genes (Supplementary Table S3). A total of 105 genes were enriched in the biological processes GO term “regulation of neuron death” (enrichment score 1.57; false discovery rate, FDR = 0.0179; p-value after Bonferroni correction for multiple testing 0.00175); and 220 genes were enriched in the GO term “chromatin organization” (enrichment score 1.41, FDR = 0.00443, p = 0.00118). The region on chromosome X together with all neighbours of this region harbour 7200 genes (Supplementary Table S4); 580 genes were enriched in the GO cellular component term “neuron part” (enrichment score 1.17, FDR = 0.00191, p > 0.05); 514 genes were enriched in GO biological processes term “response to oxygen-containing compound” (enrichment score 1.2, FDR = 0.0317, p > 0.05).

**Figure 1:**
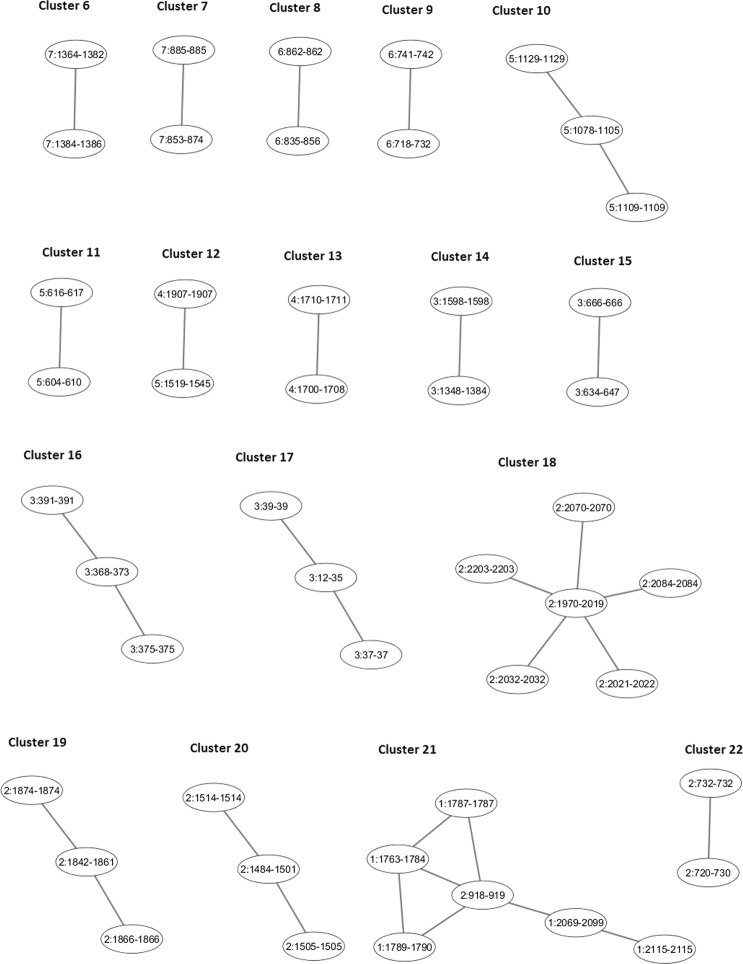
Small (12, 21) and isolated (6–11, 13–20, 22) clusters identified in the extended network of interaction.

A total of 31 SCZ-associated genes were found to reside within 13 disconnected subnetworks, present both in the original and extended networks, with approximately 2% (2176) of all SNPs residing within these genes. Although none of these SNPs reached the genome-wide significance level as recorded in the expanded PGC dataset used, recent studies^18^ performed on a bigger cohort of patients/controls identified significant SNPs in regions comprising ten disconnected subnetworks (clusters 7–9, 12–13, 15, 17–19 and 22 in Fig. 1 and Supplementary Table S5).

### Communities in the extended network

**Figure 2:**
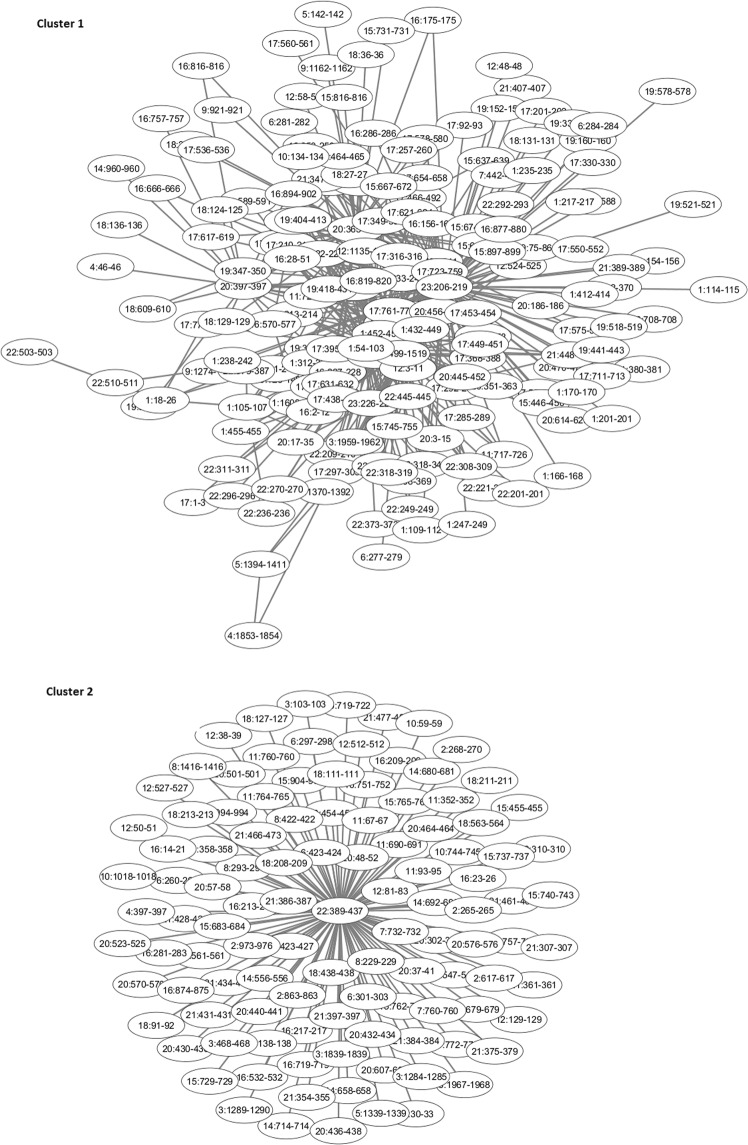
Clusters 1 and 2 identified in the extended network of interaction.

**Figure 3:**
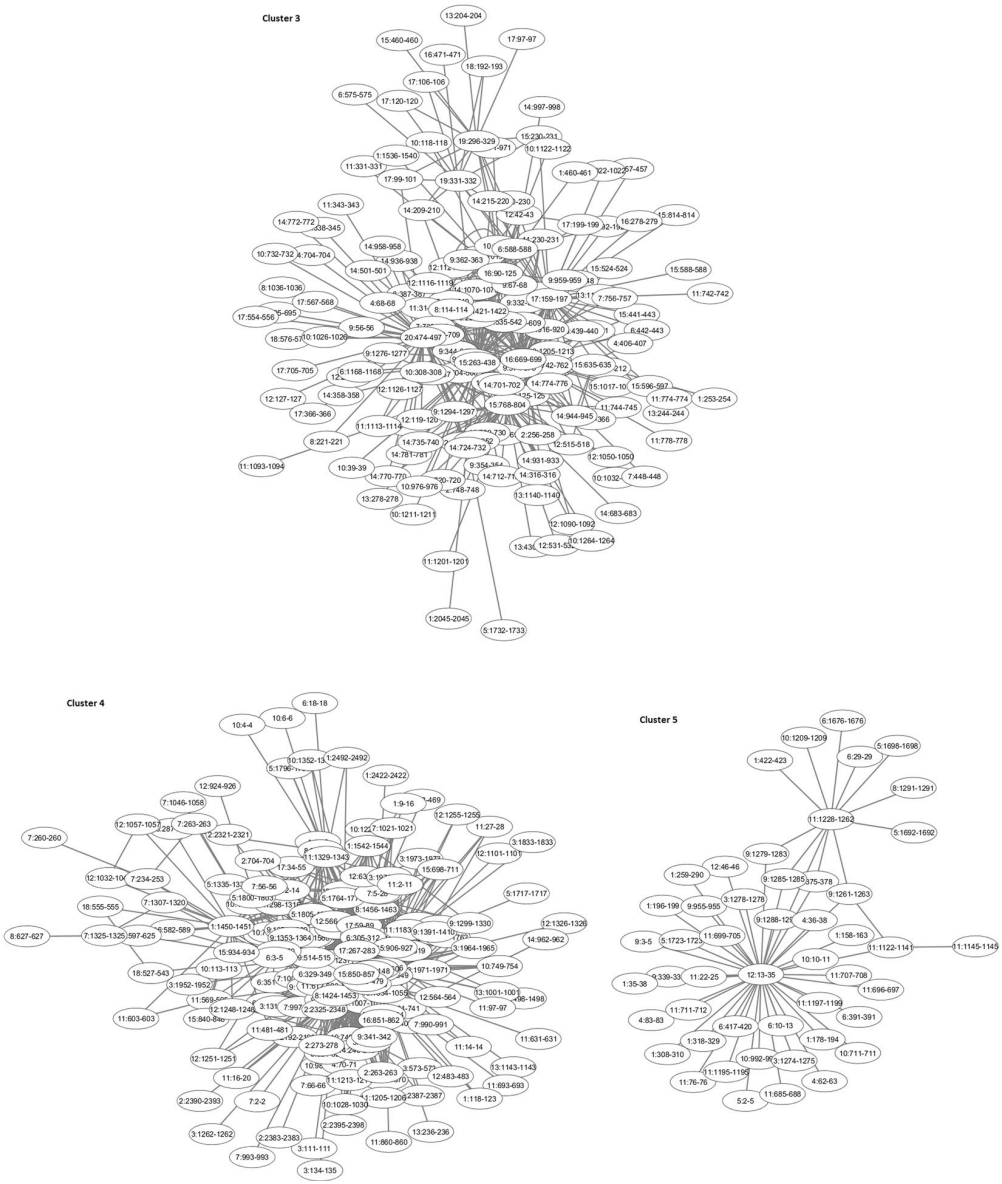
Clusters 3–5 identified in the extended network of interaction.

We identified seven communities (clusters) in the giant component of the extended network (Figs. 1–3). Larger communities have been further subdivided into smaller subcommunities (e.g. subcommunity 4 of community 1 denoted as 1.4 and so on) with the aim to find more densely connected and co-located subnetworks of genes enriched in a specific biological function and explore the role these functionally-related genes may play in the observed phenotype. A tabular representation of communities together with complete sets of genes residing within nodes comprising each community is given in Supplementary Table S5. The number of nodes in the selected communities/subcommunities and genes enriched in various GO terms are summarised in Table 1.

**Table 1:** Number of nodes in the selected communities/subcommunities and list of genes enriched in GO terms, identified by Gene Ontology Resource (GOR) and DAVID. Genes present in PGC GWAS dataset are shown in bold.

Community	Total number of nodes/length (Mb) in (sub)community	Enrichment in GO term^†^	p-value^‡^	List of genes
1	176/110.9	Alzheimer disease-presenilin pathway (GOR)	2.24 × 10^−2^	*WNT8A*, *NOTCH3* (**S**,**N**,**P**) ^§^, *MMP9* (**AD**, **S**,**N**,**P**), *PCSK4*, ***CTNNA1***, *JUP*, *TCF3*, *HN1*, *FZD2* (**P**), *WNT9B*, *MMP25*, *MMP24* (**P**), *WNT3* (**PD**,**N**), *ERN1*, *LRP5*, *LRP3*, *PSENEN* (**AD**), *KAT7*, *ERN2*, *ACTG1*, ***APH1A***, *MMP25*, *APH1B* (**AD**,**N**), *APBB3*
1.1	5/3.7	oxygen transport (GOR)	1.48 × 10^−2^	*HBA2*, *HBA1*, *HBZ*, *HBM*, *HBQ1*
1.2	40/51.3	homophilic cell adhesion via plasma membrane adhesion molecules (GOR)	3.73 × 10^−21^	*BSG*, *CDH22*, *CLSTN1*, *MYOT*, ***PCDHA1***, ***PCDHA10***, *PCDHA11*, *PCDHA12*, *PCDHA13*, ***PCDHA2***, ***PCDHA3***, ***PCDHA4***, ***PCDHA5***, ***PCDHA6***, ***PCDHA7***, ***PCDHA8***, ***PCDHA9***, *PCDHAC1*, *PCDHAC2*, *PCDHB1*, *PCDHB10*, *PCDHB11*, *PCDHB12*, *PCDHB13*, *PCDHB14*, *PCDHB15*, *PCDHB16*, *PCDHB18P*, *PCDHB2*, *PCDHB3*, *PCDHB4*, *PCDHB5*, *PCDHB6*, *PCDHB7*, *PCDHB8*, *PCDHB9*, *PCDHGA1*, *PCDHGA10*, *PCDHGA11*, *PCDHGA12*, *PCDHGA2*, *PCDHGA3*, *PCDHGA4*, *PCDHGA5*, *PCDHGA6*, *PCDHGA7*, *PCDHGA8*, *PCDHGA9*, *PCDHGB1*, *PCDHGB2*, *PCDHGB3*, *PCDHGB4*, *PCDHGB5*, *PCDHGB6*, *PCDHGB7*, *PCDHGC3*, *PCDHGC4*, *PCDHGC5*, *PVRL2*
		vasopressin synthesis pathway (GOR)	9.03 × 10^−7^	*HN1*, *MMP24* (**P**), *LRP5*
		Neuropeptide protein class (GOR)	3.05 × 10^−3^	*OXT* (**P**), *APITD1*, *PDYN* (**S**,**N**,**P**), *PPY*, *PYY (P)*, *CORT*, *AVP* (**P**), *GHRH* (**N**,**P**)
1.3	31/22.2	histone protein class (GOR)	6.32 × 10^−8^	*HIST1H2BN*, *HIST1H2AI*, *HIST1H2BM*, *HIST1H2AM*, *HIST1H2AL*, *HIST1H2AI*, *HIST1H2AK*, *HILS1*, *HIST2H2AC*, *HIST2H2BC*, *HIST1H2AJ*, *HIST1H2BL*, *HIST1H1B*, *HIST2H2AA3*, *HIST2H2AA4*, *HIST2H2BE*, *HIST2H4A*, *HIST2H4B*, *HIST1H4K*, *HIST1H4L*, *HIST1H4J*, *HIST1H2BO*, *HIST2H2AB*
		histone fold (DAVID)	4.6 × 10^−12^	*HIST1H2AI*, *HIST1H2AJ*, *HIST1H2AK*, *HIST1H2AL*, *HIST1H2AM*, *HIST1H2BL*, *HIST1H2BM*, *HIST1H2BN*, *HIST1H2BO*, *HIST1H3H*, *HIST1H3I*, *HIST1H3J*, *HIST1H4J*, *HIST1H4K*, *HIST1H4L*, *HIST2H2AA3*, *HIST2H2AA4*, *HIST2H2AB*, *HIST2H2AC*, *HIST2H2BC*, *HIST2H2BE*, *HIST2H3A*, *HIST2H3C*, *HIST2H4A*, *HIST2H4B*
		DNA methylation (DAVID)	6.4 × 10^−7^	*ARID3B*, *DNMT1*, *ELAVL1*, *GNGT2*, *KANK2*, *KLF1*, *LASP1* (**P**), *RAB11B*, *RAB3D*, *SETDB1*, *SNF8*, *SRCIN1*, *SMARCE1*, *WIPF2*, *ADGRL1*, *C17orf96*, *CARM1*, *CCDC117*, *CPLX3* (**P**), *DIDO1*, *DOCK6*, *EEF1A2*, *HNRNPM*, *HIST1H1B*, *HIST1H2AI*, *HIST1H2AJ*, *HIST1H2AK*, *HIST1H2AL*, *HIST1H2AM*, *HIST1H2BL*, *HIST1H2BM*, *HIST1H2BN*, *HIST1H2BO*, *HIST1H3H*, *HIST1H3I*, *HIST1H3J*, *HIST1H4J*, *HIST1H4K*, *HIST1H4L*, *HIST2H2AA3*, *HIST2H2AA4*, *HIST2H2AB*, *HIST2H2AC*, *HIST2H2BC*, *HIST2H2BE*, *HIST2H3A*, *HIST2H3C*, *HIST2H4A*, *HIST2H4B*, *ILF3*, *MED15* (**S**,**P**), *MRPL4*, *MYH11*, *MYL4*, *NFIX*, *NCOA3*, *PALM3*, *RPRD2*, *SEMA7A*, *ZNF687*
2	110/28.6	DNA demethylation (GOR)	3.47 × 10^−2^	*APOBEC3D*, *APOBEC3A*. *APOBEC3B*, *APOBEC3C*, *APOBEC3F*, *APOBEC3H*, *APOBEC3G*
		histone protein class	1.2 × 10^−7^	*HIST1H2BH*, *HIST1H1A*, *HIST1H2AC*, *HIST1H2AD*, *HIST1H4G*, *H2AFY2*, *HIST1H1C* (**PD**,**N**), *HIST1H2AE*, *HIST1H2AB*, *HIST1H4A*, *HIST1H4B*, *HIST1H4H*, *HIST1H4C*, *HIST1H4D*, *HIST1H4E*, *HIST1H4F*, *HIST1H1D*, *HIST1H2BI*, *HIST1H2BF*, *HIST1H2BE*, *HIST1H2BG*, *HIST1H2BC*, *HIST1H1T*, *HIST1H2BD*, *HIST1H1E*, *HIST1H2BB*
3	154/45.5	lipase protein class (GOR)	2.6 × 10^−2^	*PLD6*, *PLA2G4E*, *CES3*, *LIPC* (**AD**,**N**,**P**), *CES2*, *PLA2G1B* (**S**,**P**), *PLA2G4D* (**P**), ***LCAT*** (**N**,**P**), *CES4A*, ***PLA2G15***, *PLA2G4B*, ***PLCB2*** (**S**,**N**,**P**), *PLA2G4F*
4	164/135.1	flavonoid glucuronidation	FDR = 4.18 × 10^−2^	*UGT1A3*, *UGT1A4*, *UGT1A1*, *UGT1A5*, *UGT1A7*, *UGT1A10*, *UGT1A8*, *UGT1A6*, *UGT1A9*
		vasopressin synthesis pathway (GOR)	2.25 × 10^−3^	*NEU1*, *SEC. 11 A*, ***SPCS1***, *NEU2*
5.3	11/5.3	Olfactory receptor	1.2 × 10^−14^	*OR10G4*, *OR10G7*, *OR10G8*, *OR10G9*, *OR10S1*, *OR4D5*, *OR6M1*, *OR6T1*, *OR6X1*, *OR8A1*, *OR8B12*, *OR8B2*, *OR8B3*, *OR8B4*, *OR8B8*, *OR8D1*, *OR8D2*, *OR8D4*, *OR8G1*, *OR8G2*, *OR8G5*
21	6/5.9	regulation of immune response	1.62 × 10^−2^	*CD34*, ***CR1L***, *IL10* (**AD**,**PD**,**S**,**N**,**P**), *MAPKAPK2*, *CR1*, *C4BPB*, ***CD46***, *CD55*, *PIGR*, *CR2* (**PD**,**N**), *C4BPA*
		regulation of complement cascade	8.3 × 10^−5^	*CR1*, *C4BPB*, ***CD46***, *CD55*, *CR2* (**PD**,**N**), *C4BPA*

^†^Only a fraction of GO terms is listed;

^‡^all p-values are corrected for multiple testing: Bonferroni correction in Gene Ontology Resource and Benjamini in DAVID;

^§^**AD**, **PD**, **S**, **N**, or **P**, next to the specific gene, indicates its association with Alzheimer’s, Parkinson’s or schizophrenia disease, neurological or psychological disease class, respectively, as identified by DAVID software.

From the genes residing within regions comprising five largest components (clusters 1–5) of the extended network, several groups of genes with significant enrichment in GO terms associated with various biological processes, pathways, reactome pathways and protein classes emerged. The highest fold enrichment (FDR < 0.05) was observed for “regulation of complement cascade” (31.81), “DNA demethylation” (13.43) and “DNA methylation” (10.72) (see Table 1).

## Discussion

In this study we used a new network-based approach for identification of novel genes/loci associated with a specific phenotype that takes into account the co-location and shared biological function of newly found genes with so-called “guilty” genes, i.e. genes that are known to associate with the phenotype in question. Using schizophrenia as a case study we compiled a dataset of 346 “guilty” genes identified by early PGC GWAS^6,7^ but expanded with genes/SNPs identified via a comprehensive literature search. In the resulting dataset, 212 of “guilty” genes were in common with the recently compiled lists of high-confidence and SCZ risk genes^19^ (http://resource.psychencode.org). Moreover, 58 loci were found to be in common between loci harbouring 346 genes and 145 loci identified by a recent meta-analysis as harbouring significant SCZ-associated SNPs^18^.

Using non-brain specific Hi-C interaction data for the GM12878 cell line, which preserves ~70% interactions recorded in adult brain cells that have recently become available via PsychENCODE resources^19^, we created an original network of interactions between regions harbouring those 346 “guilty” genes and extended it by adding regions that have strong inter-chromosomal interactions with the nodes/regions from the original network. All the 143 loci identified in ref. ^18^ were present in the extended network. Out of the 302 and 606 genes listed, respectively, in high-confidence and SCZ risk gene lists^19^, 80% and 70% of genes were found to reside within the regions comprising the extended network created using non-brain Hi-C data. These rather high overlaps show the potential of the proposed approach to identify novel SCZ-related genes. Note that our approach is solely based on co-location and shared biological function of newly found genes with the “guilty” genes. These percentages, on the other hand, may reflect the fact that only 70% of brain-specific interactions are preserved in the karyotypically normal B lymphoblastoid cell line GM12878 used in this study.

In several examples below, we discuss the plausibility of genes/SNPs residing within various important (from network science point of view) nodes or clusters found contributing to SCZ either individually or selectively. In the absence of individual genotype data, the assessment of cumulative effect of several SNPs is purely speculative and needs further investigation.

### Important nodes

Two nodes in the extended network corresponding to regions chr22:38,800,001–43,700,000 and chrX:20,500,001–21,900,000 were found to be of possible importance on the basis of the high degree (number of interactions with other regions) of their corresponding nodes. The latter region together with all connected regions harbours 7200 genes (Supplementary Table S4) enriched in several GO terms. For example, 580 genes were enriched in the GO cellular component term “neuron part”. Among these genes there were 21 genes (e.g. *CHRNA2*, *CHRNA5*, *CACNA1C* and *CACNA1D*) from the SCZ high-confidence gene list^19^ and 26 genes (e.g. *CHRNA3*, *CLU*, *GRIN2A*, *MAPK8IP1* and *PARL7*) from the SCZ risk gene list^19^. One of the novel genes found in this group is flotillin 1 gene, *FLOT1*, located on chromosome 6. This gene is highly expressed in the brain and harbours 15 SNPs with one of them, rs1059612, reaching genome wide significance level (p = 1.03 × 10^–8^) and another one, rs1064627, having p-value just below significance level (p = 8.39 × 10^−7^). Recent studies^18^ involving larger datasets did not show a strengthened association of these SNPs with SCZ. Nevertheless, the STRING interaction network provided by GeneCard (https://www.genecards.org/), shows protein-protein interactions of FLOT1 protein with LRFN3 and CORO1C proteins. Our analysis shows that these genes also occur within close proximity to the *FLOT1* gene, i.e. all three of them interact with chrX:20,500,001–21,900,000 region and are present in the set of 580 genes enriched in “neuron part” GO term although none of them are present in SCZ high-confidence or risk gene lists^19^. The leucine rich repeat neuronal 3 gene, *LRFN3*, although not present in the expanded PGC dataset and the recent GWAS datasets^18^, has been found to be associated with schizophrenia and severe progressive autism by other GWAS (https://www.genecards.org/). One can speculate that existence of protein-protein interactions, close proximity of these genes within the nucleus and shared biological function, makes the *FLOT1* gene (as well as other genes from this set of 580 genes) a potential candidate, linked to schizophrenia. SNPs residing within these genes, not necessarily meeting genome-wide significance threshold, may still cause the phenotype, but either collectively, i.e. together with other SNPs residing within these co-located genes, or selectively when SNPs in different functionally-related genes could show significant association with the phenotype in a smaller subgroup, e.g. in a subgroup of patients with both schizophrenia and severe progressive autism. Note that the absence of *CAV1*, *SCC6A3* and *SVIL* genes, which exhibit protein-protein interaction with FLOT1, from the resulting dataset of genes is likely to be due to our stringent selection of inter-chromosomal interactions by considering only 0.05% of the strongest interactions, recorded in Hi-C datasets.

### Disconnected subnetworks

The occurrence of SNPs in genes residing within isolated subnetworks could indicate their association with a specific manifestation of schizophrenia in groups of patients stratified by e.g. schizophrenia sub-phenotypes. Although none of the SNPs residing within 31 SCZ-associated genes comprising 13 disconnected subnetworks reaches the genome-wide significance level in the expanded PGC dataset used, several later studies showed their significant association with SCZ. Fromer *et al*.^20^ have identified five single-gene loci with three of them ‒ *CNTN4*, *CLCN3* and *SNAP91* ‒ residing respectively within subnetworks 17, 13 and 8 (Fig. 1, Supplementary Fig. S2 and Table S5) with the remaining two genes, *FURIN* and *TSNARE1*, residing within cluster 4 (see Fig. 3 and explanation below). Fromer *et al*.^20^ have shown that some SNPs in these genes could alter the expression of these genes in patients with schizophrenia. All these genes were later found to harbour significant SNPs^18^. In addition, our analysis of the extended network shows that the promoter of the *CNTN4* gene (cluster 17 in Fig. 1 and Supplementary Fig. S2), which is a member of the contactin family of immunoglobulins and plays a role in the development of nervous system, has strong interactions with the promoter regions of the *LRRN1* gene (node labelled 3:39–39 in subnetwork 17); nine SNPs residing within this promoter region could also contribute to the change in expression of the *CNTN4* gene. The *LRRN1* gene itself, which was not in our expanded PGC dataset and is not present in the recent set SCZ-associated genes^18^, was reported to have an altered RNA expression in blood of patients with Alzheimer’s disease^21^. Another gene, the synaptosome associated protein 91 *SNAP91*, was present in the expanded PGC dataset harbouring 75 SNPs (albeit with p-values ≥ 7.86 × 10^−4^). It was later shown^18^ that it harbours a SNP rs217331 (p = 1.17 × 10^−12^). The promoter of this gene has a strong intra-chromosomal interaction with the *NT5E* gene promoter region not previously found to associate with SCZ. Garcia-Esparcia *et al*.^22^ found that the 5′-nucleotidase gene, *NT5E*, is involved in purine metabolism and deregulated in Parkinson’s disease brains. These two examples demonstrate that SNPs in these co-located genes could be important in smaller subgroups of SCZ patients also suffering either from Alzheimer’s or Parkinson’s disease. One can also speculate about a possible existence of a shared genetic mechanism underlying several psychological and neurodegenerative disorders.

Interestingly, some of the SNPs found in genes residing within the isolated subnetworks are associated with specific manifestation of schizophrenia and, possibly, of a different sub-phenotype. For example, the *MAN2A1* gene residing within an isolated cluster 10 (Fig. 1 and Supplementary Fig. S2) was previously found to associate with SCZ (p = 3.05 × 10^−08^) but was discarded from the list of SCZ-associated genes based on the results of meta-analysis (see ref. ^18^ and Supplementary Table 5 therein). This particular gene was found to associate with eye movement dysfunction in schizophrenia^23^ and may not show significant association with SCZ when patients are not stratified by sub-phenotypes; the occurrence of this gene within an isolated subnetwork may indicate its association with specific manifestation of schizophrenia.

### Clusters

Nine genes in cluster 4 (Fig. 3) were found to be enriched in the “flavonoid glucuronidation” GO term (FDR = 4.18 × 10^−2^). None of these genes were present in the expanded PGC dataset but were reported elsewhere as associated with SCZ. Among them are the *UGT1A1* and *UGT1A4* genes (UDP glucuronosyltransferase family 1 member A1 and A4), which harbour 20 and 58 SNPs, respectively, with p-values above 4.1 × 10^−2^. Erickson-Ridout *et al*.^24^ have shown that two polymorphisms in these genes significantly alter glucuronidation of the antipsychotic drug Clozapine, used for treating refractory schizophrenia. These polymorphisms were found to be important in determining inter-individual differences in drug metabolism *in vivo*. Other newly found genes within this cluster, also involved in glucuronidation and co-located with the two known genes, could provide new screening targets and help to determine the right dosage, patient response and side effects to either Clozapine or other drugs being developed for treating schizophrenia.

In total 716 genes, residing within cluster 2 (Fig. 2) and including the histone cluster gene family (prefix *HIST1*) genes, were enriched in the “histone” protein class and “DNA demethylation” (Table 1). Four of these genes (*HIST1H2BD*, *HIST1H2BC*, *HIST1H2BH* and *HIST1H2BG*) form a part of the major histocompatibility complex (xMHC) region on chromosome 6. Several GWAS have shown its strong association with schizophrenia (see, for example, ref. ^25^). Using whole genome gene expression profiling, Sanders *et al*.^26^ identified a set of 95 differentially expressed transcripts enriched in immune-related genes, listed above. Other genes from the extended xMHC, e.g. *HIST1H4C*, *BTN3A3* and *ZNRD1* (also in cluster 2), were also found as potential schizophrenia genes^27^. The latter harbour a SNP with p-value meeting the genome-wide significance level (p = 8.80 × 10^−9^). Recent results of meta-analyses of CLOZUK and PGC GWAS data^18^ showed the improved significance of SNPs in this region (12997 SNPs in total, p = 4.32 × 10^−44^ for the index SNP rs1233578). Note that the primary function of the family of histone genes is to facilitate the organisation of DNA on chromatin. Hence, SNPs affecting these genes may disrupt the local 3D architecture of the human genome either collectively, when several genes are disrupted in the same patient, or individually if SNPs show high association with a given phenotype, thus perhaps resulting in the SCZ phenotype as reported in ref. ^27^. One can speculate that other genes from the histone cluster gene family found in this community (see Table 1) could also contribute to schizophrenia risk.

In addition, two out of 511 SNPs residing on chr6:25,900,001–26,600,000 region in cluster 2, rs3857547 (chr6:26157762; p = 1.46 × 10^−9^) and rs7749823 (chr6:26158079; p = 6.01 × 10^−9^), reach genome-wide significance level of association with schizophrenia. ENCODE data indicates that these SNPs occur within an enhancer/promoter, targeting several genes including *HIST1H2BD*, *HIST1H1E*, *BTN2A2*, *BTN2A1*, *BTN2A3P*, *BTN3A1*, *BTN3A2* and *BTN3A3* also residing within cluster 2 and therefore located in close proximity to each other within the cell nucleus. It is possible that this enhancer/promoter, found in several cell lines and tissues including H1 neuronal progenitor and GM12878, could target other remotely located genes residing within this cluster.

DNA methylation has been implicated in the etiopathology of various complex disorders. Several studies have examined DNA methylation changes in schizophrenia. For example, rs2114724 (not present in the expanded PGC dataset) and rs2228611 SNPs in the DNA methyltransferase 1 gene, *DNMT1*, increase the risk of developing schizophrenia in males or are associated with early onset of schizophrenia and with family history in South Indian population^28^. Although cluster 2 was not found to be enriched in “DNA methylation” GO term, six genes of the butyrophilin family (*BTN3A2*, *BTN2A3P*, *BTN2A1*, *BTN3A3*, *BTN3A1*, *BTN2A2*) were found to reside within this cluster. Correlation between DNA methylation and gene expression in these four genes, including the butyrophilin gene *BTN3A3*, found in patients with SCZ and bipolar disorder was reported in ref. ^29^. One can speculate that DNA methylation of the butyrophilin genes correlates with their expression in a way, similar to the *BTN3A3* gene^29^; their methylation patterns could be novel candidate factors conferring risk to schizophrenia in patients either with bipolar disorder or an early onset of schizophrenia.

Six genes ‒ *CR1*, *C4BPB*, *CD46*, *CD55*, *CR2*, *C4BPA* ‒ in cluster 21 were enriched in the “regulation of complement cascade” GO term. Håvik *et al*.^30^ demonstrated the significant role of complement control-related genes in the etiology of schizophrenia and support disease mechanisms that involve the activity of immunity-related pathways in the brain. Several common SNPs in the complement control-related gene *CSMD2* and its homolog *CSMD1* and in the complement surface receptor *CD46* residing in cluster 21 have exhibited an association with SCZ across three large samples comprising 1133 patients and 2444 healthy controls^30^, although the p-values obtained by recent meta-analysis^18^ no longer show significant association. According to the DAVID database, the complement C3d receptor 2 gene, *CR2*, has been also found in association with Parkinson’s and other neurological disorders. Further analysis of other complement control-related genes found within this cluster is required to prove their association with SCZ. Interestingly, the association of the major histocompatibility complex locus with SCZ was partly explained by structurally diverse alleles of the complement component 4 (C4) genes^31^. As a result, varying levels of C4A and C4B expression in the brain are generated, implicating excessive complement activity in the development of schizophrenia that leads to the reduced numbers of synapses in the brains of individuals with schizophrenia.

Three genes in cluster 1.2 ‒ *HN1*, *MMP24*, *LRP5* ‒ were enriched in the “vasopressin synthesis” pathway (Table 1). Vasopressin and oxytocin were found to be associated with cognitive and clinical symptoms severity in midlife women with chronic schizophrenia^32^. The DAVID database also shows an association of the *MMP24* gene with Parkinson’s disease. These observations show that although SNPs in these three genes do not exhibit genome-wide significance, they may still play an important role, but in a smaller well-defined subgroup of patients. In the same vein, down-regulation of the olfactory receptors, co-located in cluster 5.3, in the dorsolateral prefrontal cortex in patients with chronic schizophrenia was noted by Ansoleaga *et al*.^33^.

Cluster 1.3 was enriched in the “DNA methylation” GO term (DAVID, p = 6.4 × 10^−7^ after Benjamini correction), “histone” protein class (GOR, p = 6.32 × 10^−8^) and “histone fold” (DAVID, p = 4.6 × 10^−12^). The latter set of 25 genes is a subset of 60 genes enriched in the “DNA methylation” GO term (Table 1). Eight out of 122 SNPs residing within the region chr6:27,600,001–27,900,000 – rs34706883 (chr6:27805255; p = 5.07 × 10^−10^), rs13212651 (chr6:27806985; p = 6.2 × 10^−10^), rs17693963 (chr6:27710165; p = 1.56 × 10^−10^), rs13194781 (chr6:27815639; p = 5.45 × 10^−10^), rs13199772 (chr6:27834085; p = 7.05 × 10^−10^), rs17749927 (chr6:27669976; p = 1.01 × 10^−9^), rs17750424 (chr6:27701122; p = 1.49 × 10^−9^) and rs13218875 (chr6:27884012; p = 1.02 × 10^−8^) – reached genome-wide significance level for association meaning that each of these SNPs may cause the observed phenotype, but in different patients. Moreover, according to the ENCODE data the first two SNPs that reside within enhancer/promoter regions target various genes in the histocompatibility complex region as well as remotely located glutathione peroxidase 5 and 6 genes, *GPX5* (chr6:28,493,789–28,502,728) and *GPX6* chr6:28,471,073–28,483,570), known to protect cells and enzymes from oxidative damage. These two genes were also found to associate with SCZ^34^. Interestingly, the region harbouring these SNPs may form looping interactions with the promoters of the *GPX5* and *GPX6* genes; a strong intra-chromosomal interaction is recorded between these regions in Capture Hi-C data. Expression of other genes residing within this community could be also influenced by SNPs found on chr6:27,600,001–27,900,000.

Five genes from the haemoglobin subunit gene family (*HBM*, *HBA2/HBA1*, *HBZ*, *HBQ1*) located on chr16:100,001–900,000 in cluster 1.1 were enriched in the “oxygen transport” GO term. The role of genes of the haemoglobin family is to transport oxygen throughout the body via red blood cells; alterations in these genes could prohibit a sufficient amount of oxygen from entering the brain, which is a cause known to increase the risk of SCZ in adults^35^. The emerging role of haemoglobins, as a potentially novel mechanism underlying mental disorders, was discussed by Altinoz & Ince^36^.

Twenty-four genes residing within cluster 1 were enriched in the “Alzheimer disease (AD)-presenilin pathway”. Several genes from this cluster were also implicated in neurodevelopmental disorders and schizophrenia. Prenatal viral-like immune activation in a mouse model displayed long-term epigenetic modification in Wnt-signalling genes *Wnt3* and *Wnt8a* in adult offspring^37^, prompting a plausible explanation for the disruption of prefrontal gene transcription and behavioural functions in patients with prenatal infectious histories. Passos Gregorio *et al*.^38^ studied the link of non-synonymous variants in five genes of the Notch-signalling pathway ‒ *NOTCH2*, *NOTCH3*, *JAGGED2*, *ASCL1* and *NUMBL* ‒ to schizophrenia in two independent and unrelated populations. The authors found that only SNPs in the *NUMBL* gene, which also resides within cluster 1, have an association with schizophrenia. The *ASCL1*-harbouring locus was also one of the new loci, identified in ref. ^18^. Gao *et al*.^39^ reported a specific abnormality in peripheral expression of the matrix metalloproteinase 9 gene, *MMP9*, and its methylation, indicating a potentially different pathological mechanism underlying a specific subgroup of schizophrenia, which includes deficit schizophrenia patients. In turn, gene expression profiling of the dorsolateral and medial orbitofrontal cortex in schizophrenia patients^40^ identified seven novel genes, including *KAT7* and *EVI2A*, and previously reported gene *TARDBP* (all residing within cluster 1) that are differentially expressed in several brain regions, including prefrontal cortical regions, in patients with SCZ. Fazzari *et al*.^41^ have demonstrated the physiological role of Aph1b-γ-secretase (*APH1B*) in brain and its relevance to schizophrenia. We speculate that enrichment of SCZ genes identified via network analysis, which takes into account the proximity of genes within the cell nucleus, in “Alzheimer disease-presenilin pathway” GO term may imply that both AD and schizophrenia share a common genetic risk at least for some specific phenotypes, e.g. deficit schizophrenia as reported in ref. ^39^.

A finer structure of intra-chromosomal interactions was explored by analysing interactions within extended gene regions. For example, the EGR region on chr17:400,001–32,00,000, residing within community 4.1, harbours 58 genes, including four genes, *TSR1*, *SGSM2*, *SMG6* and *SRR*, that were present in the expanded PGC dataset. The *SRR* gene, which encodes an enzyme that synthesizes D-serine from L-serine, was involved in a co-expression module associated with schizophrenia disease status^42^. The authors have also shown that a SNP, rs16952025 (p = 0.47 in the expanded PGC dataset), residing ~90 K upstream of the *SRR* gene within an intron of the neighbouring *SMG6* gene, was significantly associated with the expression level of the *SRR* gene. We found that the region harbouring this SNP has a strong intra-chromosomal interaction with the *SRR* gene promoter; it is plausible that this SNP occurs within an enhancer that controls the *SRR* gene expression via chromatin looping interaction. Although no association with the expression of the *SMG6* gene itself was found^42^, the association study performed by Tabarés-Seisdedos *et al*.^43^ on schizophrenia and bipolar patients from a Spanish isolated population found an association between structural variant in the *SMG6* gene and prefrontal cognitive deficit in patients with SCZ and bipolar disorder. In the subsequent studies^18^, the locus harbouring the *TSR1*, *SGSM2*, *SMG6* and *SRR* genes was also found to be significantly associated with SCZ. Two genes, *SMG6* and *SRR*, were also found as genes strongly associated with SCZ^19^.

Two nodes in a two-node isolated subnetwork 12, connecting regions chr5:151,800,001–154,500,000 and chr4:190,600,001–190,700,000, was looked at in greater details. The region on chromosome 5 harbours 18 genes and 1264 SNPs (the smallest p = 7.23 × 10^−6^ for rs12522297), including the *GRIA1* and *GALNT10* from the expanded PGC dataset harbouring 221 and 181 SNPs with p-values above 2.75 × 10^−4^, respectively. No genes were found in the region on chromosome 4 (UCSC Genome Browser, GRCh37/hg19; http://genome-euro.ucsc.edu). Fourteen SNPs were found in this region; none of them have reached genome-wide significance level (p ≥ 5.36 × 10^−4^). Interestingly, this region harbours 161 transcription factor binding sites (TFBS), identified by ChIP-Seq technology. It is commonly accepted that transcription factors that bind to TFBS either activate or repress the transcription of the target gene(s) that usually reside in the close proximity of the TFBS. With the new patches, available for hg19 assembly, only one RNA gene – long intergenic non-protein coding RNA 1262 (*LINC01262*) ‒ was found within the adjacent 100 Kb region upstream of the TFBS-enriched regions. Diseases associated with *LINC01262* include late-onset of Parkinson’s disease (https://www.genecards.org/). Several genes/pseudogenes (*DUX8L8*, *DUX4L6*, *DUX4*, *DUX4L4*, *DUX4L5*, *TUBB4Q* and *FRG1/2*) were found more than 200Kb downstream of the TFBS-enriched regions. The contraction of the macro-satellite repeats in DUX-pseudogenes and centromeric deletion involving the *FRG1* gene were reported as a cause of muscular dystrophy in general and facioscapulohumeral muscular dystrophy in particular. No disorders were recorded in GeneCards for tubulin beta 7 pseudogene *TUBB7P* and the *LINC01596* RNA gene residing more than 200Kb downstream of the TFBS-enriched regions. One can speculate that some of the target genes may reside within strongly interacting region, chr5:151,800,001–154,500,000, and TFBS influence the expression of one or several of the 19 genes residing within this region.

In conclusion, this study provided the evidence for using co-location of genes/genomic regions governed by the 3D architecture of the human genome for predicting genes and genomic regions linked to a specific phenotype. Using our network approach, several novel candidate regions were found to harbour genes with prior reported or recently confirmed associations with SCZ, acting as a proof of concept. The use of networks of intra- and inter-chromosomal interactions for relevant cell lines or tissues, coupled with the information on the enrichment of respectively coding and non-coding regions in various schizophrenia-related GO terms and functional elements pave the way for understanding the contribution of seemingly remote SNPs/genes to polygenic disorders, such as SCZ, and provide a plausible biological explanation for their cooperation. This approach therefore promises to be of potential utility in identifying novel genes/genomic regions linked to other polygenic disorders and the means of aggregating genes/SNPs according to their co-location and shared biological function for further investigation, e.g. by using polygenic scores.

## Materials and Methods

### Dataset of SNPs used in this study

In this study we further expanded a dataset of SNPs downloaded from the Psychiatric Genomics Consortium website https://www.med.unc.edu/pgc/results-and-downloads/downloads (permission was obtained in 2012) by SNPs found via review of literature and the use of various bioinformatics resources. The resulting dataset comprised 1,252,901 SNPs spanning 22 chromosomes. Variants on chromosome X were not considered. Each SNP has an accompanying summary statistic that includes a p-value, showing the strength of association with SCZ. The p-values lie within the range 4.3 × 10^−11^ < p < 0.998 with only 136 SNPs reaching the genome-wide significance threshold of p < 5 × 10^−8^. A total of 346 genes with putative association with SCZ, identified by the PGC^6,7^ and other studies, were used. We refer to these datasets of SNPs and genes as expanded PGC datasets. When required, genomic positions of SNPs and genes were ‘lifted over’ to the hg19 assembly using the Lift Genome Annotation program available at https://genome.ucsc.edu/cgi-bin/hgLiftOver.

### Inter- and intra-chromosomal interaction data

At the time when this study was initiated, inter- and intra-chromosomal interaction data were available for nine human cell lines, including karyotypically normal B-lymphoblastoids (GM12878), lung fibroblasts (IMR90) and mammary epithelial (HMEC). Interaction frequencies between 100 Kb regions (bins) and bins ranging from 1 Kb to 100 Kb for respectively inter- and intra-chromosomal contacts generated by *in situ* Hi-C method (FDR < 10%)^10^ were downloaded from http://www.ncbi.nlm.nih.gov/geo/ (accession number GSE63525). Henceforth, we refer to this data as *in situ* Hi-C data. Normalisation coefficients, accounting for biases introduced by experimental procedure and intrinsic properties of the human genome, were also available alongside raw counts. In this study we used inter-chromosomal contact frequencies, normalised using inter-chromosomal Knight and Ruiz normalisation procedure. In addition, a dataset of intra-chromosomal interactions between approximately 22,000 gene promoter regions and their potential enhancers, and between gene promoters of various genes obtained using the Capture Hi-C method^11^, were downloaded from http://www.ebi.ac.uk/arrayexpress/experiments/ (accession number E-MTAB-2323). Only significantly interacting regions filtered from a background noise using a one-sided cumulative binomial test and Benjamini-Hochberg correction for multiple testing with FDR < 0.05 were recorded in these datasets^11^.

### Brain expression quantitative trait loci data

The dataset of brain-related expression quantitative trait loci (eQTL) was downloaded from the Brain Expression Quantitative Trait Loci (eQTL) Almanac (Braineac)^12^. This dataset contains SNPs affecting the expression of target genes in ten distinct brain regions: the cerebellar cortex (CRBL), frontal cortex (FCTX), hippocampus (HIPP), inferior olivary nucleus (sub-dissected from the medulla; MEDU), occipital cortex (OCTX), putamen (PUTM), substantia nigra (SNIG), temporal cortex (TCTX), thalamus (THAL) and intralobular white matter (WHMT). Each SNP in this dataset is paired with a target gene in *cis* or in *trans* and an associated p-value. Small p-values correspond to a more profound target gene expression change in a given brain tissue and therefore indicate a stronger affinity to being associated with psychotic disorders. An expression averaged across all ten regions (aveALL) is also available within Braineac^12^.

### Selecting cell line to best emulate 3D interactions in brain tissues

To determine which cell line most closely resembled interactions in brain tissues, brain eQTL data^12^ was used. To align the *in situ* intra-chromosomal Hi-C data^10^ with the eQTL data, each *cis*-eQTL pair positions of SNPs and their targets were uniquely binned into regions in multiples of 10 Kb, 100 Kb and 1 Mb if the distances between SNPs and their targets were <100 Kb, <1 Mb and >1 Mb, respectively. All pairs for which both a SNP and its target occurred within the same 10 Kb bin were deleted. If several SNPs residing in the same bin were targeting the same gene, only one *cis*-eQTL pair with the lowest p-value, indicating significant change in expression, was retained. For each of the three cell lines (GM12878, IMR90 and HMEC), the number of *cis*-eQTL pairs that exhibit strong interactions (or, in other words, are nearby in 3D) was counted. The cell line exhibiting the highest enrichment of intra-chromosomal interactions in brain-related eQTL was used in subsequent analyses.

### Extended gene regions (EGRs)

Capture Hi-C data^11^ was used to identify potential regulatory regions by considering all intra-chromosomal regions strongly interacting with promoters of each of the 346 SCZ-associated genes. A *promoter-other_significant_interactions* dataset was used to identify potential enhancers, whilst promotor regions of other genes interacting with a given gene promoter and potentially influencing its expression were identified from *promoter-promoter_significant_interactions* dataset. Promoter and enhancer boundaries were calculated from start and end positions of each interacting pair of fragments; a genomic boundary was then drawn encompassing all overlapping fragments that belong to the same gene promoter or enhancer. Positions of genes and their associated regulatory regions were binned into regions in multiples of 100 Kb. In cases where several 100 Kb regions harbouring genes and/or their regulatory elements were adjacent to each other, they have been amalgamated into a longer extended gene region (EGR), sometimes spanning several 100 Kb bins and comprising several genes. The intra-chromosomal interactions between all non-overlapping and non-adjacent EGRs, promoter- and enhancer-harbouring regions were preserved (see Supplementary Fig. S3).

### Network approach to identify schizophrenia candidate regions

First, for a set of non-overlapping extended gene regions of 346 SCZ-associated genes and regions harbouring promoters and enhancers (as defined above), a network was created where nodes representing these regions were connected by edges if a *strong* inter-chromosomal interaction between at least one pair of 100 Kb fragments constituting these regions was recorded in Capture Hi-C data. All intra-chromosomal interactions identified earlier (see Extended gene regions section) were added to the network. Normalised inter-chromosomal contact frequencies between 100 Kb fragments exceeding a predefined threshold were used to measure the strength of the interactions, which were inversely proportional to their closeness within the cell nucleus. Thus, the resulting network, henceforth called the *original network*, comprises nodes corresponding to the extended regions of the 346 SCZ-associated genes and/or their regulatory regions and possible interactions between these regions that arise due to their close spatial proximity within the cell nucleus.

Next, new nodes and edges were added to the original network by including all 100 Kb regions that have strong (above a predefined threshold) inter-chromosomal interactions with the nodes from the original network, or in other words, by adding first nearest neighbours to the nodes of the original network. All adjacent regions have been amalgamated into longer regions, and links between amalgamated nodes were added (or kept) if at least one 100 Kb fragment from these longer regions interacted with 100 Kb fragments constituting either another 100 Kb or an amalgamated region. We refer to this network as the *extended network*.

To identify important nodes within the network, for each node its *degree* was calculated as a number of connections a node has. The choice of this specific measure was largely predefined by the way networks were created by considering only first nearest neighbours of the SCZ-associated regions. In addition, the Girvan‒Newman fast greedy algorithm^44^ implemented as GLay plugin^45^ (Su *et al*., 2010) to Cytoscape^46^ was used to detect communities within the resulting network, i.e. groups of nodes/regions that are densely connected to each other within a given community but sparsely connected to nodes in other communities of the network.

### Additional bioinformatics resources used to investigate candidate regions

Upon identifying SCZ candidate regions (with some of them being >100 Kb long) using the aforementioned network approach, various bioinformatics resources were used to narrow down the length of regions found and to identify novel genes/loci that have the potential to be associated with schizophrenia. After compiling sets of genes residing within nodes/candidate regions, which belong to a certain network community, the enrichment of the resulting sets of genes in various gene ontology (GO) terms have been sought.

Three independent gene ontologies, compiled by the Gene Ontology Consortium^14,15^ and available at http://www.geneontology.org, together with the Database for Annotation, Visualisation and Integrated Discovery (DAVID)^16^ were used to assess the enrichment of a given set of genes in biological processes, molecular functions and cellular components. In addition, DAVID was used to identify whether any gene from a given set of genes was linked to two disease classes (neurological and physiological) and a certain disease (schizophrenia). Only GO terms with high enrichment scores and low p-values (p < 0.05 after correction for multiple testing) were considered.

## Supplementary information

Dataset 1

Dataset 2

Predicting novel genomic regions linked to genetic disorders using GWAS and chromosome conformation data – a case study of schizophrenia.

## Data Availability

List of 346 genes with putative association with SCZ, identified by the PGC^6,7^ and via an extensive literature search is given in Supplementary Table S1. List of extended genomic regions that do not interact with any other loci is given in Supplementary Table S2. Genes residing within nodes chr22:38,800,001–43,700,000 (degree 328) and chrX:20,500,001–21,900,000 (degree 304) and connected regions are listed in Supplementary Tables S3 and S4, respectively. Complete sets of genes residing within nodes, comprising each community, are given in Supplementary Table S5.
