# Supplementary material for: Predicting novel genomic regions linked to genetic disorders using GWAS and chromosome conformation data – a case study of schizophrenia
Source: Sci Rep. 2019 Nov 29;9:17940. doi: 10.1038/s41598-019-54514-2 (PMC6884554; doi:10.1038/s41598-019-54514-2)
Supplement: Supplementary file 3 — Predicting novel genomic regions linked to genetic disorders using GWAS and chromosome conformation data – a case study of schizophrenia. [file 41598_2019_54514_MOESM3_ESM.docx]

**Predicting novel genomic regions linked to genetic disorders using GWAS data and 3D architecture of the human genome – a case study of schizophrenia**

Daniel S. Buxton, Declan J Batten, Jonathan J. Crofts and Nadia Chuzhanova

School of Science and Technology, Nottingham Trent University, Clifton Lane, Nottingham NG11 8NS, UK.


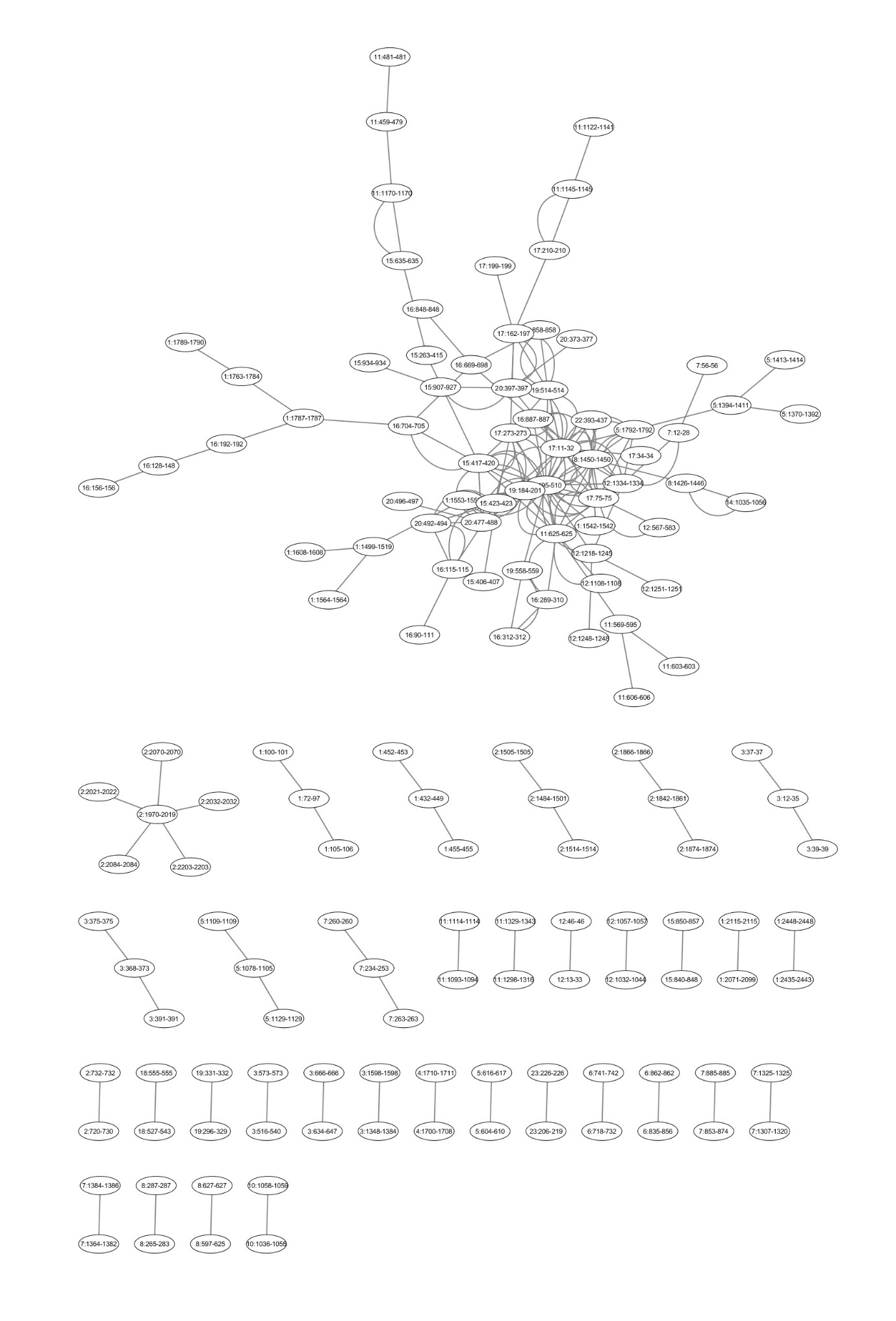


**Figure S1**. Original network of interactions between regions harbouring SCZ-associated genes.


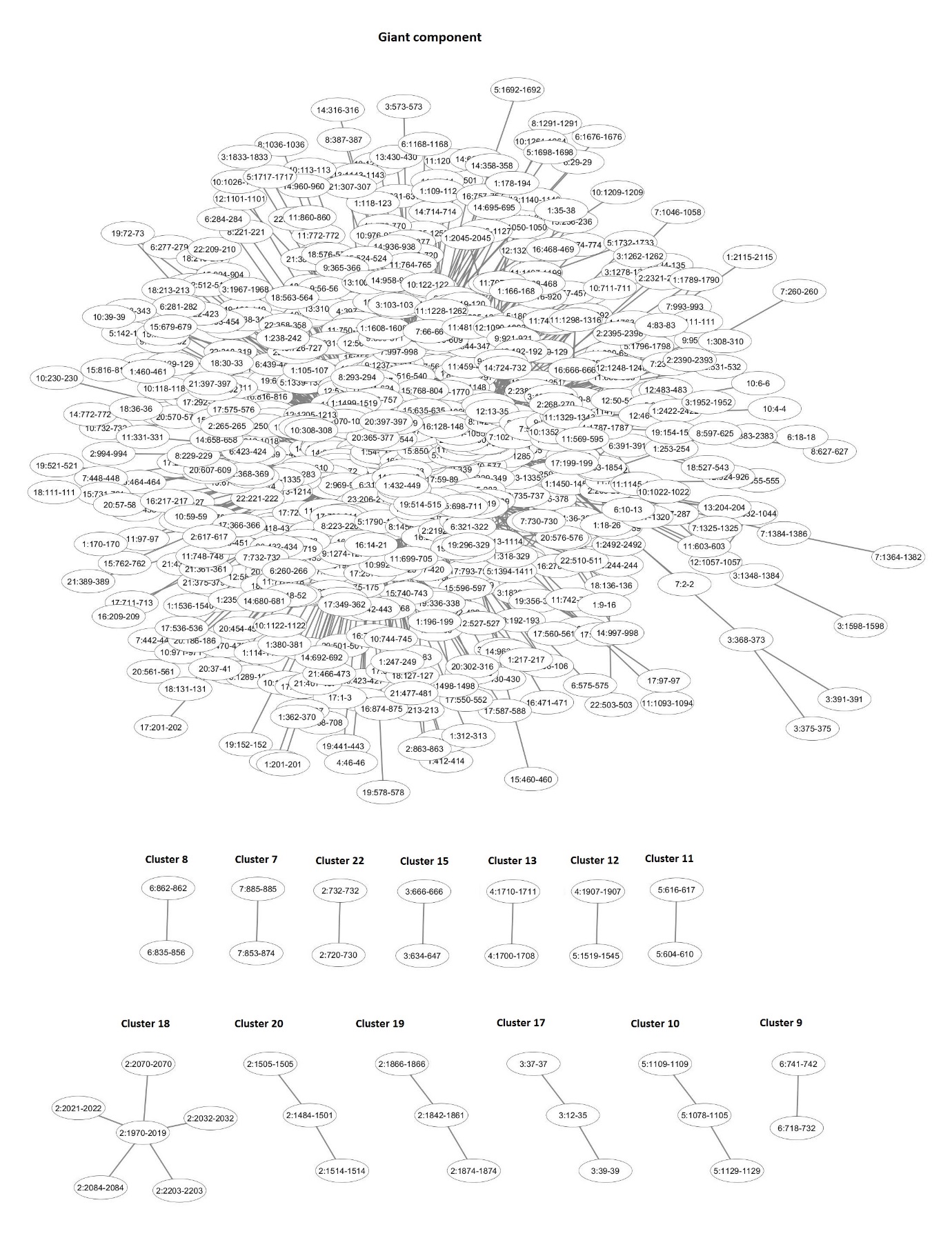


**Figure S2**. Extended network of interactions.


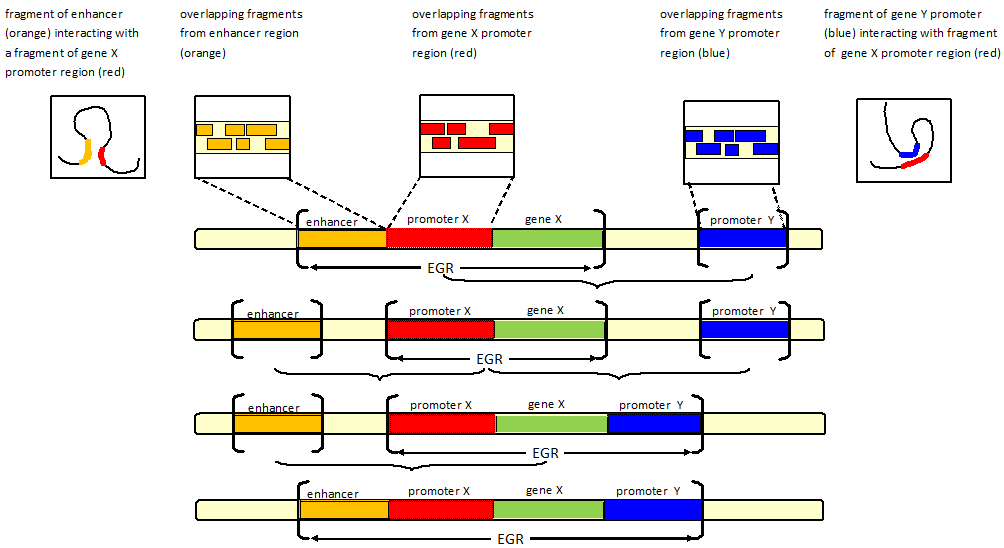


**Figure S3**. Schematic representation of a process of creating extended gene regions (EGRs) using Capture Hi-C data depending on the regions' overlap. Intra-chromosomal interactions between regions are shown by curly brackets.
